# HOPPER: implementation of a home-based prehabilitation programme with app support for patients undergoing colorectal cancer surgery—a study protocol

**DOI:** 10.1136/bmjopen-2025-104649

**Published:** 2025-11-09

**Authors:** Anne Pannekoek, Thomas Timmers, Rudolf Bertijn Kool, Hermien Schreurs

**Affiliations:** 1 Department of Surgery, Amsterdam UMC Location VUmc, Amsterdam, The Netherlands; 2 Department of Surgery, Noordwest Ziekenhuisgroep, Alkmaar, The Netherlands; 3 Amsterdam Public Health Research Institute, Amsterdam, The Netherlands; 4 Interactive Studios, Radboud University Nijmegen, ’s Hertogenbosch, The Netherlands; 5 IQ Health Science Department, Radboud University Medical Center, Nijmegen, The Netherlands

**Keywords:** Colorectal surgery, Gastrointestinal tumours, Exercise, Telemedicine, Implementation Science

## Abstract

**Abstract:**

**Background:**

Preoperative prehabilitation programmes for patients with colorectal cancer have been shown to reduce complications and hospital length of stay. However, supervised weekly physiotherapy is expensive and timeconsuming for both healthcare professionals and patients, leading to suboptimal implementation of prehabilitation programmes. A previous study demonstrated that offering home-based prehabilitation through an app is feasible and leads to outcomes comparable to supervised prehabilitation programmes. This study was conducted at a single hospital. To expand this promising modality nationwide, it is essential to identify key implementation determinants. We therefore initiated this multicentre study involving a more diverse and heterogeneous patient population. The findings of this study will provide valuable input for scaling strategies for prehabilitation programmes in the Netherlands and beyond.

**Methods:**

In this prospective multicentre cohort study, approximately 300 patients with colorectal cancer scheduled for curative surgery will be enrolled over 12 months. The study adopts a hybrid type 3 design, reporting clinical outcomes while exploring implementation-related factors. Five hospitals with varied profiles (academic, non-academic teaching and general hospitals) and geographical locations (urban and rural, high and low socioeconomic areas) are participating. The primary endpoint is the identification of barriers and facilitators, using both qualitative (interviews) and quantitative (user statistics, questionnaires) data from stakeholders. Secondary endpoints include fitness and clinical outcomes such as complications and mortality.

**Ethics and dissemination:**

This study was approved by the METC (Medisch Ethische Toetsings Commissie / Dutch Medical Ethics Committee) and was established not to apply to the Medical Research Involving Human Subjects Act (WMO / Wet Medisch Wetenschappelijk Onderzoek met mensen); submission was 21 April 2025. The app is proven safe and feasible in earlier studies and is CE certified (Conformité Européenne). Informed consent will be obtained from all patients (Supplement 1). Adverse events will be monitored and reported. Only researchers will have access to the final dataset. Results will be disseminated through publications, patient group briefings and implementation feedback to healthcare workers. Plans for sharing deidentified individual clinical trial participant-level data consist of quotes from interviews held on stakeholders. This study protocol adheres to the SPIRIT guidelines.

**STRENGTHS AND LIMITATIONS OF THIS STUDY**

- This is a hybrid study design: exploring implementation factors while measuring clinical outcomes.
- Including a heterogeneous patient population from five hospitals.
- Use of an app previously shown to be both feasible and effective.
- Identification of actual barriers and facilitators for implementation, rather than relying on assumptions.
- Conducting a hybrid study design may lead to methodological challenges requiring careful coordination and planning.

## Introduction

### Background

 In the Netherlands, the incidence of colorectal cancer in 2019 was 12 907 patients. This incidence is projected to increase by 11% by 2032, reaching approximately 14 306 cases.^1 2^ Five-year survival for stage I colorectal cancer in the Netherlands is 95%. The majority of patients (70–80%) are diagnosed with stage I–III disease and eligible for curative surgery.^3^ Perioperative care in these patients is well investigated, with protocols such as the Enhanced Recovery After Surgery (ERAS) protocol reducing complication rates and hospital length of stay.^4^ Preoperative multimodal prehabilitation—including physiotherapy, nutritional counselling, psychological support and cessation of smoking and alcohol consumption—has also demonstrated efficacy in improving fitness preoperatively and postoperatively.^5 6^ Recent studies showed improved functional capacity and muscle strength after surgery, for patients who underwent prehabilitation versus no prehabilitation.^7 8^ However, supervised weekly physiotherapy preoperatively is expensive and time-consuming for healthcare workers and patients. Not all patients participate in the prehabilitation programme due to these factors.^9^ Recent studies show that digital-supported home-based prehabilitation is feasible, equally effective and more cost-efficient. This home-based prehabilitation includes multimodal prehabilitation using timely information with push notifications.^10^

### Aims

Previous results using the app in prehabilitation provide a solid foundation for implementing this existing app in other hospitals, with the possibility of incorporating hospital-specific practices and population characteristics into the app.^10^ Our goals are twofold: first, to identify barriers and facilitators for digital supported at-home prehabilitation using the app. The purpose of this study is to expand the digital supervision on a larger scale. To develop a national implementation strategy, it is crucial to implement the concept from the previous study in Northwest Clinics in several centres, including a heterogeneous patient group as possible. This includes factors such as education level, socioeconomic status and language differences. With this scaling strategy, digital supervised prehabilitation can be made accessible to all patients with colorectal cancer. Second, we aim to further demonstrate its safety and clinical effectiveness as a scalable prehabilitation model for colorectal cancer patients.

## Methods

### Design

This is a hybrid type 3 implementation study. Over a 12-month period, we aim to include 50–70 patients per participating hospital, depending on the size of the hospital, aiming for a total of 300 patients across five hospitals. This is not based on a sample size calculation for patients, as the aim of this study is not to obtain statistical generalisability. A previous study using the same app, performed in a tertiary hospital, included 50 patients over a 12-month period. The sample size of interviewed stakeholders depends on data saturation of the answers. One hospital is an academic centre, while four are regional teaching hospitals, including one urban hospital that serves a large population with a migration background. All hospitals are in the Netherlands. Investigating clinical outcomes as a prospective cohort study will take place, and at the same time, barriers and facilitators and other implementation-related factors will be explored.

### Study population

All patients with histologically confirmed colorectal cancer (stage T1-T4NxMx) and scheduled for curative intent surgery are eligible for inclusion. Inclusion will take place in the outpatient clinic after multidisciplinary meetings where all colorectal cancer patients are discussed. All stages of cancer, ages and fitness levels will be included, with a minimum of 3 weeks to surgery. Usually, the prehabilitation period takes 3 to 4 weeks. Patients must have an email address and a smartphone or tablet. Exclusion criteria are operable patients but unable to participate in the prehabilitation programme, based on cognitive impairment diseases such as severe dementia or physical limitations such as being bedridden. Also, patients undergoing a transanal endoscopic microsurgery will be excluded, as an anastomosis will not be created. Stakeholders such as insurance officers, managers and healthcare workers such as surgeons, case managers and nurse specialists experienced in prehabilitation for colorectal cancer patients are also involved.

### Intervention

**Figure 1:**
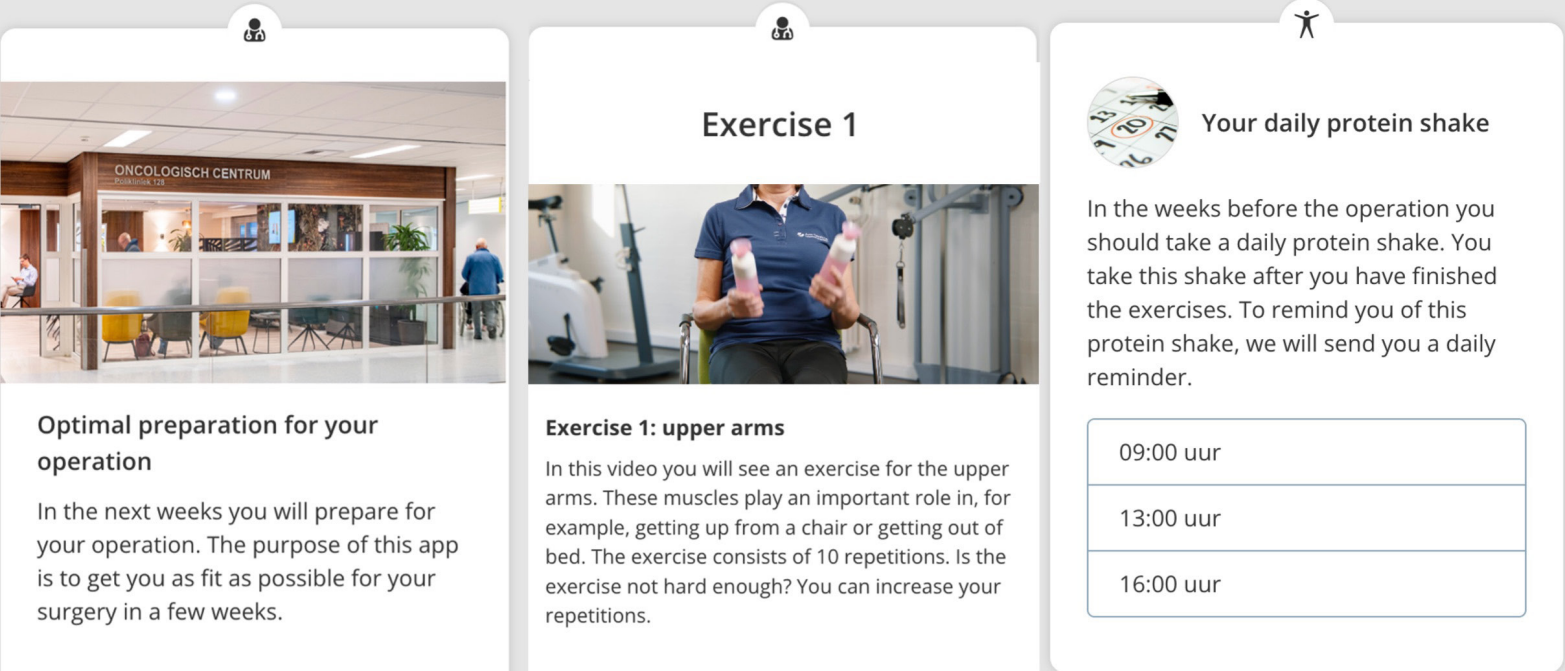
Providing timely information using the Patient Journey App.

The Patient Journey App (Interactive Studios, Netherlands) was used in the previous study in Northwest Clinics and will deliver the home-based prehabilitation programme.^11^ The app focuses on providing patients timely information (eg, exercises, nutritional advice) during the various stages of the treatment (figure 1). The app uses text, photo and video content to educate patients and to collect data via short questionnaires. The app uses push notifications to actively involve patients in their prehabilitation programme. Recruitment status is pending; anticipated enrolment of the first participating patient is 1 October 2025.

### Exploring implementation determinants: interviews

#### Qualitative data

To improve feasibility of the app, semistructured interviews will be performed on various stakeholders. The selection of interviewed stakeholders will be made by the research team. The interviews will take place during the implementation of the app in these hospitals. Determinants of implementation such as barriers and facilitators will give insight to what tailored implementation strategies are needed.

#### Quantitative data

Patients will be asked to fill in a questionnaire a few days prior to the surgery, to identify these factors as well. Together with user statistics such as number of opened push notifications, the results from the questionnaire will give insight to implementation determinants. Also, the EQ 5D (EuroQol Five Dimensions) and the EORTC QLQ C-30 (European Organisation for Research and Treatment of Cancer Quality of Life Questionnaire Core 30-item) will be administered.

The perspectives of healthcare providers and patients will be incorporated into a version of the app that can be implemented in the respective hospital with adaptations to their specific settings and practices. This way, a heterogeneous patient group will be involved in the improvement of digital prehabilitation to make it accessible for all patients.

### Clinical data collection: timeline

**Figure 2:**
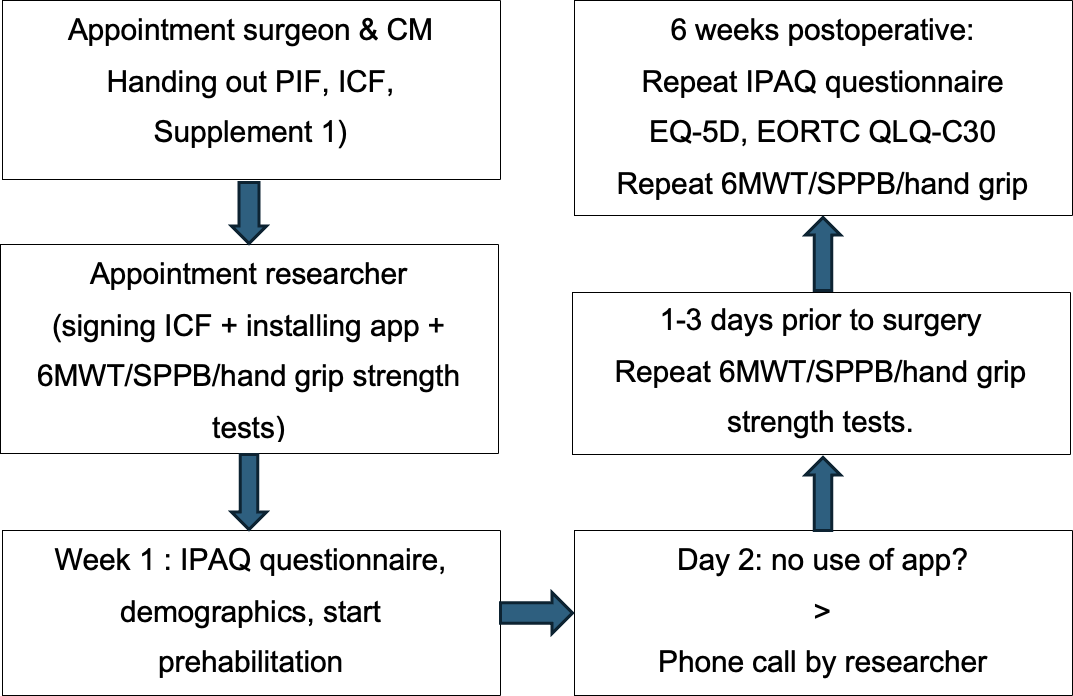
Patient’s timeline. CM: casemanager,. PIF: Patient Information Folder, ICF: Informed Consent Form, 6MWT: 6 minute walking test, SPPB: short physical performance battery, IPAQ: International Physical Activity Questionnaire.

At their appointment with a case manager or nurse specialist, information about the app and digital prehabilitation will be given. After signing the informed consent form, the patient will instal the Patient Journey App together with the researcher or nurse. At this appointment, three physical tests will be conducted by the researcher or by the nurse. These include the 6 min walking test (6MWT), the Short Physical Performance Battery (SPPB) and handgrip strength.^12^,^14^ The SPPB consists of a standing balance test, a walking speed test in 4 m and a repeated stand-chair test. In the app, the patient will fill out information according to the International Physical Activity Questionnaire (IPAQ), the Short Nutritional Assessment Questionnaire and demographic information.^15 16^ Based on the IPAQ results, patients will be categorised into an easy, medium or heavy exercise group. These exercise groups are developed by oncologic physiotherapists. Few days before the surgery, the patient will return to the hospital to repeat the 6MWT, SPPB and hand grip strength tests. Six weeks postoperatively, during their appointment with the surgeon, the three physical tests will be repeated for the last time (figure 2). The IPAQ will be repeated through the app at 6 weeks postoperative as well.

### Digital supported prehabilitation content

#### Exercise

Patients will be provided with strength-increasing videos, suitable for their fitness status at the start of the programme (eg, an easy, medium or high level). They will be encouraged to do these exercises every day. The exercises include lunges, squats and mountain climbers for example. The combination of exercises is based on experience of oncological physiotherapists and focuses on improving the aerobic capacity without losing muscle weight.^17 18^ In addition, patients will be provided with breathing exercises. Patients will be encouraged by push notifications to perform light activities such as walking or cycling for at least 30 min every day.

#### Nutrition

Patients will be referred to a dietician’s outpatient clinic. Dieticians assess nutritional status and may prescribe protein supplements. Patients will receive notifications with nutritional advice multiple times during the programme (eg, protein-rich recipes, information about vitamins and alcohol-free drinks). In addition, patients can set reminders to assure compliance with protein intake.

#### Intoxications

In the first week of prehabilitation, patients will receive information on smoking and drinking alcohol cessation. Whenever a patient smokes or vapes, their case manager or specialised nurse will refer them to a professional on smoking cessation. When a patient indicates at baseline to drink one or more alcoholic drinks per day or at least six alcoholic drinks on 1 day, he or she will be referred to a professional organisation to help quitting.^19^

#### Mental health

In the first week of prehabilitation, the patient will receive a notification assessing anxiety or sleep issues, and referrals to a medical psychologist or social work are available as needed.

### Standard care

Preoperative care continues, including blood tests, improving polypharmacy and monitoring blood sugar in diabetic patients. Also, patients will receive a blood transfusion or iron infusion when indicated and frail patients will be referred to a geriatrician. Each of the five hospitals uses various questionnaires and tests estimating frailty and regarding psychological evaluation. Estimating frailty, the most used questionnaires are the Groningen Frailty Indicator and the Identification of Seniors At Risk; for psychological evaluation, the most used tests are the Patient Health Questionnaire-9 and Hospital Anxiety and Depression Scale. These tests are mostly performed by a case manager, before referral to a geriatrician or psychologist. All patients undergo the ERAS protocol in the participating hospitals.

### Study endpoints

Primary endpoints are barriers and facilitators for patients and other stakeholders to using an app for digital supported prehabilitation, gathered by interviews, questionnaires and user statistics.

Secondary endpoints are fitness metrics (6MWT, SPPD, handgrip strength) and clinical outcomes such as length of hospital stay, complications and readmissions.

### Validation of the questionnaire

The questionnaire filled in by the patient a few days before surgery focuses on potential barriers to performing the physical exercises and preparing protein shakes and meals. The questionnaire is derived from validated questionnaires and will undergo face validity testing and reliability analysis (Cronbach’s alpha).

### Statistical analyses

The transcripts of the interviews will be coded and analysed thematically using ATLAS.ti.^20^ Questionnaire responses and secondary endpoints will be presented in absolute numbers and percentages, using 95% CIs and SD. Continuous variables will be tested using a paired t-test or the Wilcoxon signed-rank test, depending on the distribution of variables. For nominal values, the Wilcoxon signed-rank test will be used; for dichotomous variables, the McNemar test will be used. A significance level of 5% will be used.

### Patient and public involvement statement

Patients and public were involved in the study design. A steering group was made, consisting of representatives of patient associations, physiotherapists, dieticians and other healthcare workers. They all contributed to the study design and content of the app. The published article with the results will be disseminated to the participating patients.

## Ethics and dissemination

The METC (Dutch Medical Ethics Committee) approved this study and was established not to apply to the Medical Research Involving Human Subjects Act (WMO), with reference approval number 2025.0398.^1^ The app is CE certified. Patients will sign informed consent after an obligated reflection period (online supplemental file 1). Data will be stored in secure locations and is accessible only to authorised personnel. Patients will be able to continue their treatment, such as neoadjuvant chemoradiation. Frail patients will be called by the hospital or researcher after a few days of starting, in agreement with the hospitals. Patients who get injured when doing exercises at home will stop the digital supported prehabilitation and be advised to continue with a physiotherapist. Adverse events will be reported and monitored. Only the researchers will have access to the final dataset. Data sharing will take place by publishing articles, by sharing results with patient groups and by giving feedback on implementation factors and strategies to healthcare workers.

## Supplementary material

10.1136/bmjopen-2025-104649 online supplemental file 1
