# Supplementary material for: HOPPER: implementation of a home-based prehabilitation programme with app support for patients undergoing colorectal cancer surgery—a study protocol
Source: BMJ Open. 2025 Nov 9;15(11):e104649. doi: 10.1136/bmjopen-2025-104649 (PMC12598944; doi:10.1136/bmjopen-2025-104649)
Supplement: online supplemental file 1 [file bmjopen-15-11-s001.pdf]

Patient Information Folder

Get Fit for Your Operation with the Help of an App

Version: 1.0

Date: [………………]

Invitation to Participate

You are being invited to take part in a research study because you are scheduled for bowel surgery. Before you decide whether to take part, it is important to understand why the research is being done and what it would involve for you.

Please read the following information carefully. Discuss it with others if you wish. Ask us if anything is unclear or if you would like more information.

Taking part in this study is entirely voluntary. If you choose not to participate, this will not affect your treatment or care in any way.

What is the Purpose of the Study?

Good preparation before surgery is important. The fitter you are before your operation, the better your chances of a quicker and smoother recovery.

We have developed an app to help patients prepare for their operation. The app offers practical tips and advice about healthy eating, rest, and exercise in the weeks before

your surgery. This study aims to assess how well the app supports patients and whether it improves recovery outcomes.

Why Have I Been Invited?

You are eligible because you are scheduled for bowel surgery at Noordwest Ziekenhuis or a participating hospital.

Do I Have to Take Part?

No. Participation is voluntary. If you decide not to take part, or to withdraw later, this will not affect your medical care or your relationship with your doctors.

If you do decide to take part, you will be asked to sign a consent form.

What Will Happen If I Take Part?

If you agree to participate:

- You will be asked to install the app on your smartphone or tablet.
- The app will provide daily tips, videos and guidance for 3–4 weeks before your operation.
- Topics include healthy, protein-rich nutrition, good sleep habits, and strength exercises.
- At the start, you will complete a brief fitness questionnaire within the app.

- Just before surgery, you will fill in a feedback questionnaire about your experience using the app.

Fitness Assessments

We will assess your fitness three times:

1. When you install the app.
2. Shortly before your operation.
3. Six weeks after your operation at your follow-up appointment.

Each assessment involves simple physical tests taking approximately 10 minutes. If you cannot attend the hospital for the pre-operative test, a researcher may be able to visit you at home.

What Are the Possible Benefits of Taking Part?

- You will be supported to prepare for surgery from home.
- Daily exercises and advice can help improve your recovery prospects.
- You will help improve knowledge about the use of digital health tools before surgery, potentially benefiting future patients.

Please note, participation requires some of your time and effort.

What Are the Possible Disadvantages?

- You will need to attend the hospital shortly before surgery for a fitness test (approximately 10 minutes).
- Using the app will require some time each day over 3–4 weeks.

What If I Want to Withdraw?

You are free to withdraw at any time without giving a reason. Any data already collected up to the point of withdrawal will still be used in the study analysis.

How Will My Information Be Kept Confidential?

We will handle your personal information in line with GDPR regulations.

- Your data will be coded with a unique number, and only the research team can link this to you.
- Data will be securely stored and encrypted.
- Identifiable data will not be used in any reports or publications.
- Data will be retained for 10 years within the hospital.

External Data Handling

The app provider, Interactive Studios (Den Bosch, the Netherlands), will securely store usage data and questionnaire responses. This organisation complies with strict privacy and data protection standards.

Informing Your GP or Specialist

If during the study we discover information important to your health, we will inform you and, with your consent, contact your GP or specialist.

Withdrawing Consent for Data Use

You can withdraw consent for data use at any time. Data collected before this point will still be used in the analysis.

Will I Be Paid for Taking Part?

No. There is no financial compensation for participating in this study.

What If There Is a Problem?

If you have concerns about any aspect of this study, please contact the research team. If you prefer, you can contact the Patient Service team:

- **Telephone:** 020-4440700
- **Email:** PAZO-VUmc@amsterdamumc.nl

If you remain dissatisfied, you may contact the Amsterdam UMC Data Protection Officer via privacy@amsterdamumc.nl or the Dutch Data Protection Authority (Autoriteit Persoonsgegevens).

Who Has Reviewed the Study?

This study has been reviewed by the non-WMO review committee of Amsterdam UMC and was judged not to fall under the Medical Research Involving Human Subjects Act (WMO).

Contact for Further Information

Drs. Anne Pannekoek, MD

Physician-Researcher in Surgical Oncology

Department of Surgery

Noordwest Ziekenhuis Alkmaar / Amsterdam UMC, location VUmc

Tel: +31 6 25328283

Email: a.pannekoek1@amsterdamumc.nl

Thank you for considering taking part.

Participant Consent Form

Get Fit for Your Operation with the Help of an App

Name of Researcher: Drs. Anne Pannekoek, MD

Please initial each box if you agree:

☐ I confirm that I have read and understood the Participant Information Folder for the above study. I have had the opportunity to consider the information, ask questions, and have had these answered satisfactorily.

☐ I understand that my participation is voluntary and that I am free to withdraw at any time without giving a reason. This will not affect my medical care or legal rights.

☐ I give permission for relevant sections of my medical records and data collected during the study to be accessed by the research team. I understand that this information will be treated as confidential.

☐ I give permission for my GP or treating specialist to be informed if information relevant to my health is discovered during the study.

☐ I give permission for the data collected about me to be kept for up to 10 years after the end of the study, in accordance with data protection regulations.

☐ I understand that anonymised data from this study may be published in a scientific journal or shared with other hospitals and researchers, but my identity will not be revealed.

☐ I give permission to be contacted in the future about potential participation in related follow-up research studies.

Participant's Name: ___________________________

Participant's Signature: ___________________________

Date: ____ / ____ / ______

Statement by Researcher

I confirm that I have provided the participant with comprehensive information about the study and have answered any questions they have asked. I will inform the participant of any new information that may affect their willingness to continue in the study.

Name of Researcher: ___________________________

Signature: ___________________________

Date: ____ / ____ / ______

Note:

The participant will receive a copy of the signed consent form and the full Participant Information Folder.
