# Supplementary material for: HOPPER: implementation of a home-based prehabilitation programme with app support for patients undergoing colorectal cancer surgery—a study protocol
Source: BMJ Open. 2025 Nov 9;15(11):e104649. doi: 10.1136/bmjopen-2025-104649 (PMC12598944; doi:10.1136/bmjopen-2025-104649)
Supplement: Supplementary file 2 [file bmjopen-2025-104649.reviewer_comments.pdf]

PEER REVIEW HISTORY

BMJ Open publishes all reviews undertaken for accepted manuscripts. Reviewers are asked to complete a checklist review form and are provided with free text boxes to elaborate on their assessment. These free text comments are reproduced below.

ARTICLE DETAILS

Title (Provisional)

HOPPER: Implementation of a HOme-based Prehabilitation Program with app support for patients undergoing coloREctal cancer surgery, a study protocol.

Authors

Pannekoek, Anne; Timmers, Thomas; Kool, Rudolf Bertijn; Schreurs, Hermien

---

VERSION 1 - REVIEW

**Reviewer**	**1**
**Name**	**Pesce, Antonio**
**Affiliation**	**University of Ferrara, Department of Medical and Surgical Sciences and Advanced Technologies "G.F. Ingrassia"**
**Date**	**18-Jul-2025**
**COI**	**None**

Thank you for the opportunity of reviewing this paper about a very interesting and promising topic.

I have some concerns that need to be clarified:

- Have the three physical tests been conducted in the presence of an oncologic physiotherapist, or only with a nurse or researcher before surgery? The SPPB test should be clarified in more detail (e.g., sit-to-stand test, gait speed, balance tests...).

- How many weeks did the prehab program last before surgery?

- Regarding psychological evaluation, were patients assessed by a trained psycho-oncologist using validated questionnaires (e.g., GAD-7, PHQ-9)?

- Please clarify adherence to ERAS protocols preoperatively, iron deficiency anaemia should be ideally managed by parenteral iron administration as well as the assessment of frailty.

- Concerning the improvement in the physical performance and functional capacity of patients with colon cancer undergoing elective colorectal resection after surgery, I suggest to update references (https://pubmed.ncbi.nlm.nih.gov/39210058/, https://pubmed.ncbi.nlm.nih.gov/38520782/).

VERSION 1 - AUTHOR RESPONSE

Reviewer: 1
Dr. Antonio Pesce, University of Ferrara
Comments to the Author:
Thank you for the opportunity of reviewing this paper about a very interesting and promising topic.
I have some concerns that need to be clarified:
- Have the three physical tests been conducted in the presence of an oncologic physiotherapist, or only with a nurse or researcher before surgery? The SPPB test should be clarified in more detail (e.g., sit-to-stand test, gait speed, balance tests...).
  - ➔ Both points are clarified in the revised manuscript. At this appointment, three physical tests will be conducted by the researcher or by the nurse. The SPPB consists of a standing balance test, walking speed test in 4 meters, and a repeated stand-chair test.

- How many weeks did the prehab program last before surgery?
  - ➔ 3-4 weeks, this was already written in the manuscript.

- Regarding psychological evaluation, were patients assessed by a trained psycho-oncologist using validated questionnaires (e.g., GAD-7, PHQ-9)?
  - ➔ This is clarified in the revised manuscript. Estimating frailty, the most used questionnaires are the GFI (Groningen Frailty Indicator) and the ISAR (Identification of Seniors At Risk), for psychological evaluation most used tests are the PHQ-9 (Patient Health Questionnaire-9) and HADS (Hospital Anxiety and Depression Scale). These tests are mostly performed by a casemanager, before referral to a geriatrician or psychologist.

- Please clarify adherence to ERAS protocols preoperatively, iron deficiency anaemia should be ideally managed by parenteral iron administration as well as the assessment of frailty.
  - ➔ All patients undergo the ERAS protocol in the participating hospitals. Also, patients will receive a blood transfusion or iron infusion when indicated and frail patients will be referred to a geriatrician.

- Concerning the improvement in the physical performance and functional capacity of patients with colon cancer undergoing elective colorectal resection after surgery, I suggest to update references (https://pubmed.ncbi.nlm.nih.gov/39210058/, https://pubmed.ncbi.nlm.nih.gov/38520782/).
  - ➔ Added as a reference.
